# Access to information and counselling – older cancer patients’ self-report: a cross-sectional survey

**DOI:** 10.1186/s12912-017-0211-9

**Published:** 2017-04-20

**Authors:** Kristin Vassbotn Guldhav, Randi Jepsen, Siri Ytrehus, Ellen Karine Grov

**Affiliations:** 1 Department of Oncology, Førde Central Hospital, Helse Førde, PO Box 1000, 6807 Førde, Norway; 2 Nykøbing Falster Hospital, Region Sjælland, Fjordvej 15, DK-4800 Nykøbing F., Denmark; 3 Faculty of Health Studies, Sogn og Fjordane University College, PO Box 523, 6803 Førde, Norway; 4 Oslo and Akershus University College of Applied Sciences Faculty of Health, Institute of Nursing and Health Promotion, PO Box 4, St. Olavs plass, 0130 Oslo, Norway

**Keywords:** Older cancer patients, Information, Counselling, Home care nursing, Cancer coordinator

## Abstract

**Background:**

An increasingly older population, improved diagnostics and treatment increase the number of older cancer survivors, thus more than 60% of those affected by cancer are over the age of 65. Symptom relief and the prevention of functional impairment are important tasks for home care nursing, considering that patients can live a long time with their cancer disease and related side effects. The aim of this study was to investigate the extent to which cancer patients over the age of 65 reported access to information and counselling from home care nursing services, including those offered by the cancer coordinator.

**Methods:**

A cross-sectional survey was used. The survey consisted of 174 cancer patients from two regions in Norway living at home (101 women; 66–92 years). The questionnaire contained questions of various sequences including information and advices given and data on access to and use of home health care services. The questions focused on the extent to which home health care services provided the following: 1) information about the disease and treatment, 2) information about consequences and complications of the cancer disease, 3) nutritional advice and 4) advice on physical activity. Demographic, clinical and organizational variables were used. SPSS program version 22 was employed to perform descriptive and inferential statistics including correlation and logistic regression analysis. For ethical reasons, patients who were dying, delirious or with presence of cognitive impairment (any kind of dementia) were excluded.

**Results:**

The results showed that a majority (67–77%) of the respondents reported low levels of information and counselling offered. Low levels represents in this study medium, small and very small degree (Likert scale). Women, those above 85 years of age and patients with a gynecological or hematological cancer diagnosis experienced less access to information and counselling. Respondents facing availability of a cancer coordinator reported significantly higher access to information about the disease and treatment (*p* = 0.03), nutritional advice (*p* = 0.04) and advice on physical activity (*p* = 0.04) compared to those who only had contact with a home health care nurse or home health care assistant.

**Conclusions:**

The results indicated that the availability of a cancer coordinator facilitated personalized information and counselling for older cancer patients.

## Background

In pace with changes in the population approaching 2030, older patients with cancer will be an increasing patient group worldwide. Indeed, approximately 60% of new cancer patients are over the age of 65 [1]. Moreover, further improvements in tailored, intensive methods such as surgery, radiation treatment and medical cancer treatment will increase the number of older cancer survivors [2]. These changes in demography, disease patterns and treatment strategies call for more proactive health care services for older cancer patients. Future attention should focus on the ability to provide satisfactory assistance and support to this group to ensure that increasing years to life, are spent in good health as far as possible [2–4]. New health reforms in Norway emphasize treatment and care to be served in the patient’s home or in local health centers, e.g. nursing homes. Additionally, most of the patients prefer to stay at home as long as possible to enhance quality of life and keep on as usual [5]. There is limited knowledge regarding how older cancer patients evaluate follow-up and access to municipal health care services, and such knowledge is required for health care planning [6].

Many older people have good health and live active lives prior to their cancer diagnoses, but others suffer from prior chronic conditions [7]. Acute and chronic side effects of cancer treatment occur more frequently among older compared to young patients [8, 9]. Several studies have documented impaired physical and psychosocial function through the entire cancer trajectory [7, 10]. Normally, the curative and palliative phases have been considered separate entities; however, modern cancer treatment offers patients adjuvant and life-prolonging treatment for many years prior to death, which limits the traditional classification. The continuum between the phases consists of grey areas that make it difficult to define when patients turn from one stage to another [11]. The burden of symptoms and the extent to which the functional impairment inhibits daily life activities vary according to the individual patient’s situation. Factors such as age, diagnosis and comorbidity can affect the ability to reach a previous condition after completed treatment [12]. Cancer treatment can cause comorbidity or worsen chronic disease [7, 13, 14], and undernourishment and nutritional risk occur frequently among older cancer patients [15–17]. Indeed, the combination of undernourishment and reduced muscular strength among older people can result in reduced treatment effectiveness, longer hospitalizations and more frequent re-hospitalizations [18]. Several studies confirm that older cancer survivors living at home report poorer health, more Activities of Daily Living (ADL)-problems, a greater tendency to fall, poorer nutrition and more frequent visits to the medical doctor than older cancer-free persons [16, 19, 20]. Thus, early and regular support to reduce or avoid complications is especially important among older cancer patients [8].

In Norway, home care nursing is a free, universal, municipal service provided based on needs assessments. The staff includes nurses, assisting nurses and employees with no formal education. One critique of home care nursing has been that older patients must relate to many different employees. Another critique has been that the services are allocated based on a very restricted definition of the needs of the patients, which leaves little room for necessary and individually tailored advice and guidance [21]. In 2012, a new health reform, the Coordination Reform, was established in Norway. The reform intends the municipal health care to take over more tasks from the specialist health care service. Reports from the home care nursing after the commencement of the reform, signals lack of available resources, both skills and labor [22]. To meet the challenges of this particular group of cancer patients, a cancer coordinator service has been established. These nurses have specialized nursing positions that provide particular services to cancer patients living at home. The cancer coordinator should secure good interaction with different parts of the health care services and help to provide the cancer patient more continuity and stability. The service is supported partly by the Norwegian Cancer Society and is thereby a supplement to ordinary home care nursing [23, 24]. However, the municipality is responsible for these cancer coordinators. There is an inequality in the offer of the services in municipalities because the cancer coordinators are not mandatory positions. The Norwegian Directorate of Health recommends that all municipalities appoint cancer coordinators by the end of 2016 [25]. In this article, we focus on two parts of the municipal health service: home care nursing and the cancer coordinator (hereafter referred to as the home health care service).

## Methods

### Aim

The aim of this study was to discover how older cancer patients living at home assess their access to information and counselling from home health care services, including 1) the extent to which older cancer patients report that home health care services have provided information about the disease, treatment, and consequences of cancer, 2) the extent to which older cancer patients report that the health care services have provided counselling on nutrition and physical activity, and 3) comparing the assessment of access to information about diagnosis, treatment, consequences, complications and counselling about nutrition and activity with socio-demographic variables (age, gender, marital status, education and home location), clinical variables (diagnosis, time of diagnosis, active treatment, functional level and comorbidity) and health service variables such as home care nursing and/or a cancer coordinator. In this study we did not assess access to information and counselling from doctors or other professionals – except nurses and cancer coordinators.

### Design

This was a cross-sectional survey using a questionnaire. The study took place from the beginning of 2013 to December 2014 as part of a larger research project funded by the Norwegian Cancer Society. The primary objective was to investigate how health care services can provide appropriate support to older cancer patients.

The inclusion criteria included being a cancer patient above 65 years of age living at home receiving home care assistance, home care nursing and/or cancer coordinator assistance from home health care services. The participants represented patients with various cancer diagnoses, in diverse stages of the disease, and were recruited from two regions in Norway: 45 from a smaller county in Western Norway and 132 from two larger regions in Eastern Norway. The participants represented both rural and urban areas. Due to ethical considerations, we excluded patients suffering from dementia or delirium and dying patients. Of the 52 questionnaires distributed in Western Norway, 45 (87%) were returned completed. In Eastern Norway, 250 questionnaires were distributed, and 132 (58%) were returned completed.

### Recruitment

We recruited respondents either from outpatient units or through nurses working in the municipalities. We distributed the questionnaire to each patient or on request, and the project coordinator visited the patient at home to offer assistance in filling in the questionnaire. Fifty-one percent (89) received assistance completing the questionnaire.

### Data collection

The questionnaire contained questions on the presence of symptoms, ADL-problems, residential conditions, social conditions and access to and use of health care services. In this study, we used survey data on access to and use of home health care services. The questions focused on the extent to which home health care services provided the following: 1) information about the disease and treatment, 2) information about consequences and complications of the cancer disease, 3) nutritional advice and 4) advice on physical activity. The questionnaire was developed from research on factors important for older cancer patients. However, for this particular study including all aspects of concern for older cancer patients were impossible. We therefore selected “Information about diagnosis”, “Information about complications and consequences of diagnosis and treatment”, “Advice on nutrition” and “Advice on physical activity” [19, 26]. We assume that knowledge and confidence about health aspects enhance the older cancer patient’s coping and satisfaction. We have in this study consider “information and counselling” as support area, although it could just as well been defined as education.

The answers were provided on a Likert Scale (1–5). Based on these options, we analyzed the data in two different ways: 1) We measured the average answers option 1–5 and 2) We dichotomized the value 1 and 2 as «high» and 3, 4 and 5 as «low». The standardized instrument from the Eastern Cooperative Oncology Group (ECOG) was used to assess functional status [27]. The ECOG instrument includes five answer choices; we dichotomized 1 as the group with normal function and 2, 3, 4 and 5 as the group with limited/poor function. Initially, education had five values, although these were combined into two levels: >13 years and ≤13 years of completed education.

### Statistical methods

Data were analyzed using the SPSS statistical program, version 22. To compare two groups, an independent *t*-test was used for continuous variables, and a chi-square test was used for categorical variables. For each *t*-test, the effect size (ES) was calculated by Cohen’s d, and values ≥ 0.40 were considered clinically relevant [28]. For each x^2^-test, Pearson’s chi-square test and Fisher’s exact test were reported when cells were less than five. A bivariate correlation test was performed to find correlations between variables. Spearman’s rank test was used because the variables were categorical. Logistic regression analysis was performed, and the results were expressed as odds ratios (OR) with 95% confidence intervals. One-way ANOVA was used to analyze possible correlations between three age groups and reported levels of access to support. A post hoc test (Tukey) was used to test which of the age groups showed significant differences. For the questionnaire, reliability test, Cronbach’s alpha showed 0.69 with a normal distribution. The significance level for all the analyses was set to *p* < 0.05.

## Results

Table 1 shows the study sample (*n* = 174). The average age was 77 years, range 66–92 years, and 59% were women. A majority received their diagnosis more than one year ago (64%), range 0–33 years. Table 2 shows how the respondents reported access to information and counselling without differentiating between the groups. The results showed that 67% reported low access to information about the disease and 74% reported low access to information about consequences and complications due to the cancer. Seventy-five percent reported low access to nutritional advice, and 77% reported low access to advice on physical activity.

**Table 1:** Demographic and clinical characteristics of older cancer patients and their assessment of access to information and advice from home health care services (*N* = 174)

Gender, *n* (%)	
Women	101 (59)
Men	63 (41)
Age, years: mean (SD), range	77.4 (7.1), 66–92
66–74 years, *n* (%)	58 (34.5)
75–84 years, *n* (%)	81 (48.2)
≥ 85 years, *n* (%)	29 (17.3)
Civil status, *n* (%)	
Married/cohabiting	91 (53)
Single	80 (47)
Education, *n* (%)	
<13 years	129 (77)
≥ 13 years	39 (23)
Norwegian region, *n* (%)	
East	129 (77)
West	45 (23)
Cancer diagnosis, *n* (%)	
Breast	10 (6)
Lung	14 (8)
Gynecological	15 (9)
Prostate	20 (11)
Hematological	21 (12)
Colon/rectum	24 (14)
Other	35 (20)
Two or more cancer diagnoses	35 (20)
Time since diagnosis, mean (SD), range, mode	4.8 year (6.6), 0–33, 1
> 1 year, *n* (%)	109 (64)
≤ 1 year, *n* (%)	62 (36)
Ongoing treatment, *n* (%)	
Yes	95 (55)
No	77 (45)
Functional level, *n* (%)	
Normal	37 (22)
Limited/poor	130 (78)
Comorbidity, *n* (%)	
Yes	89 (53)
No	79 (47)

**Table 2:** Extent of access to information and advice from home health care services (*N* = 174)

Information about diagnosis, *n* (%)	
High	47 (33)
Low	95 (67)
Information about complications and consequences of diagnosis and treatment, *n* (%)	
High	37 (26)
Low	104 (74)
Advice on nutrition, *n* (%)	
High	34 (25)
Low	105 (75)
Advice on physical activity, *n* (%)	
High	32 (23)
Low	107 (77)

**Fig. 1:**
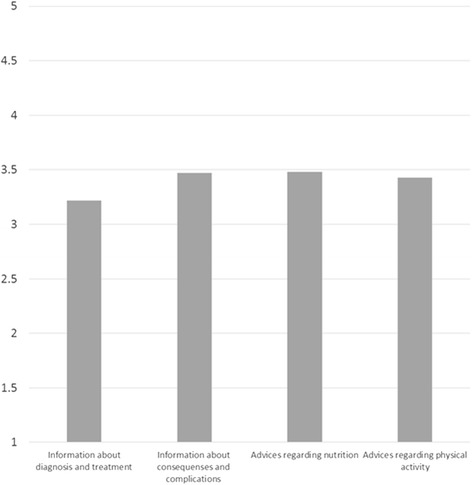
Mean extent of assessment from the home health care services for the entire sample (Likert scale 1–5)

Figure 1 shows how the respondents reported services provided in the four support areas on average. The assessed support for all areas ranged from medium (3) to low (4) by *t*-test. Table 3 shows how various groups of respondents assessed the extent of information provided, and Table 4 shows the groups’ assessment of the counselling provided. The results are reported with numbers and average ratings.

**Table 3:** Access to information about diagnosis, treatment, consequences and complications (*N* = 174)

	Information about diagnosis and treatment^a^		Information about consequences and complications^a^	
	Mean (SD)	*p*-value (ES)	Mean (SD)	*p*-value (ES)
Gender				
Women	3.33 (1.4)		3.69 (1.3)	
Men	3.07 (1.3)	0.26	3.20 (1.2)	0.03
Age				
66–74 years	3.10 (1.5)		3.46 (1.4)	
75–84 years	3.17 (1.3)		3.38 (1.3)	
≥ 85 years	3.56 (1.4)	0.35	3.75 (1.2)	0.45
Civil status				
Married/cohabiting	3.32 (1.3)		3.58 (1.3)	
Single	3.12 (1.4)	0.38	3.37 (1.4)	0.33
Education				
> 13 years	2.91 (1.2)		3.21 (1.3)	
≤ 13 years	3.12 (1.4)	0.10	3.57 (1.3)	0.15
Norwegian region				
West	3.55 (1.3)		3.75 (1.3)	
East	3.09 (1.4)	0.07	3.36 (1.3)	0.11
Cancer diagnosis				
Breast	3.86 (1.3)	0.20	4.17 (1.2)	0.19
Lung	2.92 (1.6)	0.40	3.23 (1.4)	0.50
Gynecological	3.57 (1.2)	0.30	4.23 (0.9)	0.008 (0.73)
Prostate	2.88 (1.3)	0.28	2.94 (1.1)	0.05 (0.49)
Hematological	3.89 (1.3)	0.02 (0.58)	3.94 (1.3)	0.09 (0.42)
Colon/rectum	3.35 (1.6)	0.66	3.59 (1.5)	0.69
Other	2.92 (1.3)	0.22	3.08 (1.3)	0.10
Two or more cancer diagnoses	3.03 (1.2)	0.38	3.45 (1.3)	0.94
Functional level				
Normal	3.19 (1.4)		3.48 (1.4)	
Limited/poor	3.25 (1.4)	0.83	3.49 (1.3)	1.0
Comorbidity				
Yes	3.30 (1.4)		3.49 (1.3)	
No	3.10 (1.4)	0.38	3.46 (1.4)	0.88
Time since diagnosis				
> 1 year	3.33 (1.3)		3.59 (1.3)	
≤ 1 year	3.04 (1.5)	0.23	3.29 (1.3)	0.20
Ongoing treatment				
Yes	3.16 (1.3)		3.35 (1.3)	
No	3.30 (1.4)	0.54	3.65 (1.3)	0.18
Home care nursing				
Yes	3.24 (1.3)		3.49 (1.3)	
No	3.17 (1.4)	0.80	3.41 (1.4)	0.77
Cancer coordinator				
Yes	3.04 (1.6)		3.35 (1.4)	
No	3.44 (1.3)	0.08	3.61 (1.2)	0.25

^a^Scale 1–5; higher scores represent a lower extent of access to information

**Table 4:** Access to advice about nutrition and physical activity (*N* = 174)

	Advice on nutritionª		Advice on physical activity^a^	
	Mean (SD)	*p*-value (ES)	Mean (SD)	*p*-value (ES)
Gender				
Women	3.62 (1.3)		3.74 (1.2)	
Men	3.31 (1.4)	0.26	3.03 (1.3)	0.03 (0.57)
Age				
66–74 years	3.40 (1.5)		3.38 (1.3)	
75–84 years	3.38 (1.3)		3.23 (1.3)	
≥ 85 years	3.86 (1.0)	0.26 (0.41)	4.00 (1.0)	0.03 (0.65)
Civil status				
Married/cohabiting	3.48 (1.3)		3.46 (1.3)	
Single	3.49 (1.4)	0.95	3.41 (1.3)	0.82
Education				
> 13 years	3.50 (1.4)		3.24 (1.4)	
≤ 13 years	3.49 (1.3)	0.96	3.50 (1.2)	0.29
Norwegian region				
West	3.74 (1.2)		3.49 (1.3)	
East	3.38 (1.6)	0.15	3.41 (1.3)	0.75
Cancer diagnosis				
Breast	4.00 (1.3)	0.34 (0.42)	4.17 (1.0)	0.15 (0.67)
Lung	3.08 (1.6)	0.28	2.92 (1.4)	0.14 (0.42)
Gynecological	3.79 (0.9)	0.22	4.23 (1.1)	0.02 (0.74)
Prostate	3.29 (1.4)	0.53	3.12 (1.4)	0.28
Hematological	3.68 (1.3)	0.48	3.89 (1.2)	0.09 (0.43)
Colon/rectum	3.50 (1.5)	0.95	3.38 (1.5)	0.85
Other	3.29 (1.3)	0.44	2.80 (1.2)	0.06 (0.63)
Two or more cancer diagnoses	3.58 (1.4)	0.64	3.65 (1.0)	0.29
Functional level				
Normal	3.78 (1.4)		3.63 (1.4)	
Limited/poor	3.43 (1.3)	0.23	3.39 (1.3)	0.40
Comorbidity				
Yes	3.43 (1.3)		3.55 (1.3)	
No	3.57 (1.4)	0.57	3.26 (1.3)	0.19
Time since diagnosis				
> 1 year	3.61 (1.3)		3.60 (1.3)	
≤ 1 year	3.28 (1.5)	0.16	3.15 (1.3)	0.40
Ongoing treatment				
Yes	3.46 (1.3)		3.40 (1.3)	
No	3.53 (1.4)	0.76	3.48 (1.3)	0.70
Home care nursing				
Yes	3.57 (1.3)		3.47 (1.2)	
No	3.21 (1.5)	0.16	3.31 (1.4)	0.53
Cancer coordinator				
Yes	3.28 (1.3)		3.22 (1.3)	
No	3.73 (1.3)	0.04	3.68 (1.2)	0.04

^a^Scale 1–5; higher scores represent a lower extent of access to advice

### Information about the disease and treatment

Respondents who had contact with a cancer coordinator reported statistically significantly higher access to information about the disease and treatment compared to those who did not have contact with a cancer coordinator. In contrast, those with a hematological diagnosis reported statistically significantly lower access to information about the disease and treatment than did those with other diagnoses.

### Information about consequences and complications of cancer

Women and those with gynecological cancer assessed their access to information about the consequences and complications of cancer significantly lower compared to men and those with other cancer diagnoses. Those with prostate cancer reported the highest access to information compared to the other diagnoses; this difference was close to statistically significant (*p* = 0.05) by analysis with x^2^-test. Data not shown.

### Advice on nutrition

Respondents who had contact with a cancer coordinator reported statistically significantly higher access to nutritional advice compared to those who did not have contact with a cancer coordinator. The group consisting of recently diagnosed patients reported better access to nutritional advice compared to those who were diagnosed more than a year ago. This result proved to be close to statistically significant (*p* = 0.05) by analysis with x^2^-test.

### Advice on physical activity

Women and those with gynecological cancer reported significantly lower access to advice on physical activity compared to men and those with other diagnoses. More frequently, older patients above 85 years of age reported a lower extent of advice on physical activity than the two younger age groups. We found a statistically significant difference between the group above 85 years of age and the group 75–84 years of age. The difference between the averages was small, with an effect size of 0.05. Those who had contact with a cancer coordinator reported significantly higher advice on physical activity than those who did not have contact with a cancer coordinator.

The «other» diagnosis group indicated statistically significantly better access to information about physical activity than those with named cancer diagnoses. The other variables did not show statistically significant values for any of the analyses.

## Discussion

The main finding in this study was that a majority of the elderly above 65 years of age who were all living at home reported low access to information about their disease and treatment, information about potential consequences and complications of cancer and advice on nutrition and physical activity from home health care services. It is important to emphasize that this study did not survey the needs of the older cancer patients, only their access to support; this survey investigated whether groups of older cancer patients assessed the services differently. As few previous studies have investigated patients’ assessments of this type of support from home health care services, we discuss our findings compared to other relevant studies, even if these have described various unmet needs. All studies referred to in the discussion part have informational needs included.

Several studies in recent years have documented that physical activity and proper nutrition, in varying degrees, can contribute to maintaining function and reducing afflictions from side effects of the disease and treatment [29–31]. However, early intervention is important to prevent weakening in these areas among older cancer patients, and especially among those with comorbidity where the reserve capacity may be weakened by a cancer diagnosis [7, 8, 14]. Considering that functional impairment is greatest the first year after a diagnosis [12], that older cancer patients are frequently at nutritional risk or are undernourished [17, 18] and that older people are more prone to side effects from treatment [9], it would have been preferable, from a professional perspective, to have more offers from all four support areas. Our study revealed that 78% of the respondents had limited physical function and 53% reported comorbidity; thus, it is disturbing that the offers of support in general were low for both nutritional advice and physical activity. In total, 80% of those with comorbidity reported low advice on physical activity, and between 60 and 76% similarly reported low access to information about their disease and treatment, about consequences and complications and about nutrition.

Several studies have shown that older cancer patients have different, unmet needs before, during and after active cancer treatment [6, 32–37], which underpins the need for support to this group. Older cancer patients are a heterogeneous group with varying needs depending on the diagnosis, treatment and patient’s own health.

We found no significant differences in the assessed access to information and counselling between those who were undertaking treatment and those who were not. In addition, the length of time since the diagnosis did not produce any significant differences. Amongst patients undertaking treatment, there may be both newly diagnosed patients and patients with a relapse of the disease. This was not apparent in our study, although it is likely that those who reported two or more cancer diagnoses (35%) registered a relapse. A majority of both groups received low access to support. However, at an almost significant level, differences between the time of diagnosis and nutritional advice were found such that newly diagnosed patients reported higher access to support than those diagnosed a year or more ago.

In their review of 30 studies, Puts et al. [6] found that 40–90% of older, newly diagnosed patients had a high degree of unmet needs during active treatment, including the need for information. Different survey tools, different definitions of «unmet» needs and the fact that various groups with different treatments were included in the studies can explain the differences. In only three of the studies, the average age was approximately 70 years, which represented a younger population compared to our study where the average age was 77. Harrison et al. [34] also found, in an earlier review, that the need for information was greatest during treatment. Morrison et al. [33] did not find any differences in unmet needs among those who were newly diagnosed and those who had been diagnosed earlier, or between those who received either chemotherapy and/or surgery. In the same study the respondents’ reported that need for information about their disease and treatment was satisfactorily met. This was in contrast to the two studies mentioned above by Puts et al. [6] and Harrison et al. [34]. Beck et al. [32] studied older patients living at home with an average age of 71 at one and three months after completed cancer treatment and found that symptoms continued to have effects, especially on physical function.

Jansen et al. [38], in their review of 17 studies, found that cancer patients above 65 years of age would like information about their disease and treatment, providing it is not too comprehensive and detailed. Another study shows that older people might need information repeated more than once and that they also need more tailored information than younger patients [39]. A majority of the participants in our study had received their diagnosis one year ago, and approximately 70% had received their diagnoses within the past three years. One must take into consideration that the results of this study may be biased because those who were diagnosed more than one year ago could have received information but unfortunately had forgotten. However, between 62 and 71% of the newly diagnosed reported low access to support in all four surveyed areas.

Studies of older cancer patients’ needs at 6 months [35], 14 months [36] and several years [37] after their treatment ended have shown that a majority have fewer unmet needs as time increased from the treatment and diagnosis. One common feature in these three studies was that they included small groups, which still showed unmet needs after a period of time. Here also, on average, the participants were somewhat younger than in our study, and the groups of diagnoses were more homogenous.

In this study, patients with gynecological and hematological cancer reported the lowest access to available information. Other studies have shown that these are groups with several unmet needs, especially regarding information [36, 40, 41]. The respondents with «other forms of cancer» reported the highest access to advice on physical activity. As the diagnoses included in this group will vary from study to study, it is difficult to compare the results across studies.

In this study, women and those above 85 years of age more often reported low access to advice on physical activity. Other studies have shown that women and old age are predictors of unmet needs and that women most often report unmet needs [6, 34]. However, several studies have shown that older cancer patients report fewer unmet needs than younger cancer patients [36, 38]. As revealed from the results more women than men reported lower access to information and advice. This finding could signify a bias since those with gynecological cancer was one of the groups that reported the lowest level of access – and this group comprise only women.

Information regarding diagnosis and treatment was the area with highest access to support compared to the areas of access to information about consequences and complication and counselling to nutrition and physical activity. We suggest that nurses still focus less on the patients’ response to the disease and treatment, and more on the disease since evidence based programmes on cancer treatment recently have been prepared and published [42].

In 2012, approximately 100 cancer coordinator positions were established in Norwegian municipalities, partly funded by the Norwegian Cancer Society. In their review, Steiro et al. [23] found that coordinating efforts for cancer patients can improve quality of life. This information corresponds to findings from our study, which showed that those who had contact with a cancer coordinator reported significantly higher access to information about the disease and treatment, nutritional advice and advice on physical activity compared to those who only had contact with a home health care nurse or home health care assistant.

The present study indicated a positive effect of cancer coordinators in service to older patients living at home with cancer, and this result is worth noting. A cancer coordinator will have particular knowledge of and attention to the special needs of elderly cancer patients. At the same time, we must also not ignore the time dimension because the cancer coordinator has available time in addition to the ability to provide in-depth personalized information and advice for the cancer patients and their families.

### The limitations of the study

To our knowledge, no other studies have specifically investigated older cancer patients’ self-reported access to this type of support from home health care services. Thus, one of the limitations of our study is the lack of a sufficient basis for comparison. At the same time, earlier research was focused on more nuanced context-specific study [27]. Another limitation is that this study reported results for only the healthiest patients because we excluded a number of patients due to effects from the disease and treatment. There was also an underrepresentation of older cancer patients above the age of 85, which may have contributed to an imbalance in age groups. Data from the study were derived from self-reports, and this approach represents the gold standard for research about service needs. However, the questionnaire is not psychometrically tested which is an obvious limitation.

We also offered assistance in completing the questionnaire, which might have affected the results. However, we assume that more questionnaires would have been incompletely completed without such assistance.

### Future research

In general, more research is needed on the home health care service, particularly how this service can be organized and offered to older cancer patients. It could also be interesting to ask home care nurses about what kind of information and counselling, and appropriate communication media they find most relevant for this target group. Further, it would be interesting to conduct a longitudinal study for a more nuanced survey about the possible changes in access to support from home health care services during cancer progression. A survey of the respondents’ use of home health care services prior to their cancer diagnosis could be of interest to obtain a picture of how the service changes with a cancer diagnosis. Finally, a survey of the cancer coordinator’s role for older cancer patients throughout the entire cancer trajectory is desirable.

## Conclusions

The aim of this study was to survey older cancer patients living at home on their access to information and counselling from home health care services. A majority of respondents reported having received low or moderate access to these services. According to previous research cited in this article, one must assume that the respondents had varying degrees of need for support. According to the literature, the respondents represent a vulnerable group, and because few reported a high extent of support, it is reasonable to question whether the services are sufficient and sensitive to the needs of the elderly. We have seen here that there is a slight tendency for women, those above 85 years of age and patients with a gynecological or hematological cancer diagnosis to experience less access to support. Thus, health personnel in home health care services should be particularly aware of delivering information about the disease and its consequences and advice regarding nutrition and physical activity to older cancer patients. Local and national authorities should also note that cancer coordinator availability could have a positive impact on older cancer patients living at home.

## Abbreviations

ADL: Activities of Daily Living
ECOG: Eastern Cooperative Oncology Group
